# Psychometric evaluation of a multidimensional family resource assessment tool for schizophrenia (M-FRAT) from caregivers’ perspectives in communities of Beijing, China

**DOI:** 10.3389/fpsyt.2026.1818532

**Published:** 2026-09-10

**Authors:** Ting Li, Meirong Wang, Zhaolu Pan, Lifen Chen, Guanghui Jin, Xiaoqin Lu

**Affiliations:** 1 School of General Practice and Continuing Education, Capital Medical University, Beijing, China; 2 Department of General Practice, Beijing Chao-Yang Hospital, Capital Medical University, Beijing, China; 3 Department of General Practice, Beijing Tongren Hospital, Capital Medical University, Beijing, China; 4 Department of Education, Xuanwu Hospital, Capital Medical University, Beijing, China

**Keywords:** caregiver, community, family resources, schizophrenia, psychometric evaluation

## Abstract

**Objective:**

A standardized and comprehensive instrument specifically designed to evaluate the support needs of families affected by schizophrenia is needed. This study aimed to evaluate the psychometric properties of the multidimensional family resource assessment tool for schizophrenia (M-FRAT), which was developed to assess family resources among families affected by schizophrenia from the perspective of family caregivers and to inform family-centered support strategies in primary health care.

**Methods:**

A total of 304 family caregivers were purposively recruited from seven community health service centers in Beijing. Validation was conducted in two stages. Stage 1: content validity was assessed by an expert panel using a four-point Likert scale. Stage 2: item analysis, reliability, and construct validity were evaluated using data from a caregiver survey, which included sociodemographic characteristics, the M-FRAT, and the SF-36. Item analysis was performed using critical ratio and item-total correlation analysis. Reliability was assessed using Cronbach’s alpha. Construct validity was examined through structural validity and hypothesis testing. Structural validity was evaluated using exploratory factor analysis (EFA) with supplementary principal axis factoring (PAF) and parallel analysis. Hypothesis testing included known-groups validity and associations between M-FRAT and SF-36 scores.

**Results:**

The M-FRAT comprises 37 items across seven factors, including three internal family resource domains (financial, psychological-spiritual, and medical treatment–related support) and four external domains (social, cultural, economic-environmental, and medical resources). Item-level content validity was high (I-CVI = 0.88-1.00), and all items demonstrated satisfactory discrimination and item-total correlations. The scale showed excellent internal consistency (Cronbach’s α = 0.927). EFA indicated a seven-factor structure explained 70.245% of the total variance, which was further supported by PAF and parallel analysis. The structural support dimension was removed during instrument refinements. Higher M-FRAT scores were positively associated with SF-36 domain scores, providing preliminary support for construct validity through hypothesis testing.

**Conclusions:**

The M-FRAT demonstrated good content validity, construct validity, and reliability. It may assist primary health care professionals in evaluating family resources systematically through caregiver reports, which facilitates tailored and family-centered schizophrenia rehabilitation support to reduce the caregivers’ burden.

## 1 Introduction

Schizophrenia imposes substantial and enduring burden on both the affected individuals and their families (1, 2). In many countries, particularly in Asia, most individuals with schizophrenia reside with their families (3, 4), who consequently assume the role of primary caregiver. Family caregivers of individuals with schizophrenia undertake complex and demanding responsibilities, including daily supervision, medication management, emotional support, and coordination with health and social services (2). Caregivers are exposed to multiple stressors across physical, psychological, emotional, social, and economic domains (5–7), resulting in significantly poorer quality of life (QoL) compared with caregivers of individuals with chronic physical conditions (8–10). Such strain also compromises the quality of care provided by caregivers (11), thereby hindering patients’ recovery (12, 13).

Adequate support and resources have been shown to improve caregivers’ QoL and caregiving capacity (14–16), which in turn contribute to better patient outcomes (3). Family-based interventions are widely recognized for their benefits in relapse prevention (17–19). Family resources (FRs) encompass multiple domains of support, including social, cultural, religious, economic, educational, environmental, and medical resources, which may be provided by relatives, friends, health professionals, and other supportive individuals (20, 21). Comprehensive assessment of FRs enables systematic identification of available family resources and unmet needs, thereby informing targeted family support and intervention strategies. However, existing assessment tools often focus on isolated dimensions—most often social support (e.g., the Multidimensional Scale of Perceived Social Support, Social Support Rating Scale) (22–25) or family functioning (e.g., the Family Assessment Device, the Family Adaptability, Partnership, Growth, Affection, and Resolve Index) (26–28)—and they might not be able to capture the multidimensional needs of comprehensive support for family. Moreover, few instruments are specifically tailored to the needs of families affected by schizophrenia.

A multidimensional family resource assessment tool for schizophrenia (M-FRAT) was developed by our research group based on the foundations and key principles of family resources in general practice (21). Its development followed a multi-stage process involving literature review, questionnaire survey, in-depth personal interviews, and Delphi consultation (29). The tool was designed to comprehensively assess available FRs and specific needs among families affected by schizophrenia, thereby facilitating sustained rehabilitative support within routine mental health care (29). The initial version comprised 46 items across 10 dimensions (**Supplementary Material**), including five internal FRs (financial, psychological-spiritual, medical treatment, information-education, and structural support) and five external FRs (social, cultural, economic, environmental, and medical resources). This study aimed to evaluate the psychometric properties of the M-FRAT among family caregivers in community settings in Beijing, China, to support its application in primary health care.

## 2 Materials and methods

The study adopted a sequential two-stage design: (1) assessment of content validity by an expert panel; and (2) evaluation of item reduction, reliability, construct validity, and empirical validity using data obtained from a caregiver survey.

Item exclusion was performed using a comprehensive screening strategy based on the results of item analysis, construct validity, and reliability, with detailed criteria reported in the Stage 2 statistical analysis section.

### 2.1 Stage 1: content validity

#### 2.1.1 Recruitment of participants

A panel comprising eight experts was convened to evaluate the relevance, clarity, and representativeness of each item. Eligibility criteria were as follows: (1) currently working as a mental health doctor, researcher, or general practitioner; (2) having at least two years of professional experience in the field of mental illness; (3) possessing knowledge of the management of individuals with schizophrenia; and (4) having prior experience in research projects.

#### 2.1.2 Data collection

The expert panel completed a brief questionnaire to assess the relevance of each item to the measurement objectives and the overall construct. Each item was rated using a 4-point Likert scale (1 = not relevant, 4 = highly relevant).

#### 2.1.3 Data analysis

Content validity was assessed using the item-level Content Validity Index (I-CVI) and the average scale-level CVI (S-CVI/Ave). An I-CVI value greater than 0.78 and an S-CVI/Ave value > 0.90 were considered indicative of good content validity (30, 31).

### 2.2 Stage 2: item analysis, reliability, and construct validity

#### 2.2.1 Survey sample

A two-stage sampling strategy was employed between November 2021 and February 2022. During the first stage (November-December 2021), seven community health service centers (CHSCs) were randomly selected from 24 CHSC teaching sites of Capital Medical University, and coordination with CHSC administrators and psychiatrists was undertaken to confirm site participation and train investigators before study implementation. During the second stage (January-February 2022), eligible participants were purposively recruited from the selected CHSCs based on predefined inclusion criteria. Following the guideline that the sample size should be 5 to 10 times the number of variables (32), and allowing for an anticipated 20% missing data, the minimum required sample size was 276.

The family caregivers of patients with schizophrenia were selected based on the following inclusion criteria: (1) living with the patient, taking primary responsibility for the patient’s care and fully understanding the situation of both the patient and the family; (2) being able to answer the questions clearly and logically; and (3) demonstrating a willingness to participate in the study.

#### 2.2.2 Data collection

Data on sociodemographic information, the M-FRAT, and the SF-36 were collected through the Wenjuanxing online survey platform. The SF-36 was used to examine the empirical validity of the M-FRAT. To ensure data completeness, all questionnaire items were set as mandatory, and questionnaires could not be submitted until all items had been completed.

The M-FRAT consisted of 46 items categorized into 10 dimensions (see **Supplementary Appendix I**). Each item was rated on a five-point Likert scale ranging from 1 (strongly disagree) to 5 (strongly agree). Total scores were calculated by summing item scores and ranged from 46 to 230, with higher scores indicating greater levels of family resources availability.

The SF-36 is a widely used instrument for assessing health-related QoL and has demonstrated good reliability and validity in China (33). It comprises eight domains: Physical Functioning (PF), Role-Physical (RP), Bodily Pain (BP), General Health (GH), Vitality (VT), Social Functioning (SF), Role-Emotional (RE), and Mental Health (MH). Raw scores for each domain are transformed into standardized scores ranging from 0 to 100, with higher scores indicating better QoL.

#### 2.2.3 Data management

This study was deemed exempt from ethics approval by the Medical Ethics Committee of Capital Medical University. Written informed consent was obtained from each participant involved in this study. All participant information was kept confidential. Before the survey, the study objectives and procedures were explained to the administrative leadership of the selected CHSCs, and approval was obtained. Trained psychiatrists facilitated data collection after receiving an overview of the study aims. Family caregivers were also informed about the study purpose and provided with written informed consent. Data were collected using both paper-based and electronic questionnaires. To ensure confidentiality, questionnaires did not collect any personal identifiers.

#### 2.2.4 Statistical analysis

No item-level missing data were identified in the final analytical dataset. Statistical analysis was conducted using SPSS 27.0. Descriptive analysis was used to describe caregivers’ characteristics. Continuous variables were presented as mean ± standard deviation (SD), and categorical variables were presented as frequencies and percentages.

##### 2.2.4.1 Item analysis

(1) Critical Ratio (CR) Method

Participants were ranked by their total M-FRAT scores and categorized into the top 27% (high-score) and bottom 27% (low-score) groups. Item-level differences were assessed using independent samples *t*-tests, with CR ≥ 3.0 and *P* < 0.05 indicating adequate discriminative power (34).

(2) Item–Total Correlation

Item–total correlations were assessed using Spearman coefficients (r) to evaluate the consistency of each item with the overall scale. Items with r < 0.30 were considered for potential removal. Changes in Cronbach’s α after deleting each item were also reviewed to identify items that might reduce internal consistency.

##### 2.2.4.2 Reliability

Internal consistency reliability was evaluated using Cronbach’s alpha, with a value of 0.70 or higher considered acceptable (35).

##### 2.2.4.3 Construct validity

The construct validity of the M-FRAT was examined through structural validity and hypothesis testing (36). Hypothesis testing was conducted by assessing predefined expectations regarding differences between known groups and associations with theoretically related constructs (37).

(1) Structural validity

Exploratory Factor Analysis (EFA) was conducted to evaluate the structural validity of the M-FRAT. The suitability of the data for factor analysis was confirmed by a Kaiser-Meyer-Olkin (KMO) value greater than 0.70 and a significant Bartlett’s test of sphericity (*P* < 0.05). Principal component analysis (PCA) with varimax rotation was initially performed to explore the dimensional structure of the M-FRAT. Given the relatively large number of initial items (>40), the distinction between PCA and common factor methods was considered unlikely to substantially affect the factor solution at this exploratory stage (38). The number of factors retained was based on eigenvalues greater than 1, scree plot inspection, and interpretability of the factor structure. A cumulative explained variance > 60% (38) and factor loadings ≥ 0.50 were considered acceptable criteria for interpreting the factor structure (34). To further evaluate the robustness of the identified factor structure and provide additional evidence for structural validity, common factor analysis using principal axis factoring (PAF) with promax rotation was conducted. The number of factors was further examined using FA-based parallel analysis, with factors retained when the observed eigenvalues exceeded the corresponding eigenvalues derived from randomly generated datasets.

(2) Hypothesis testing

(i) Known-groups validity

Known-groups validity was examined based on the hypothesis that M-FRAT scores would differ between urban and rural families. This hypothesis was based on the assumption that FRs available for long-term schizophrenia management are influenced by both internal family capacities and the external healthcare and social support environments. Previous studies have indicated that families affected by schizophrenia may encounter challenges in accessing healthcare and social support resources, and that the availability of primary healthcare resources may vary across residential contexts (39, 40). Therefore, urban and rural residence was considered a theoretically relevant grouping variable for evaluating known-groups validity. Differences in total M-FRAT scores between urban and rural groups were assessed using independent-samples t tests or Mann–Whitney U tests according to data distribution. Differences in domain scores were additionally explored to identify specific dimensions contributing to potential group differences.

(ii) Association between M-FRAT and SF-36 scores

Construct validity was further examined by testing the association between M-FRAT and SF-36 scores. Although M-FRAT and SF-36 assess different constructs, they are theoretically related. FRs represent an important foundation for caregivers’ adaptation to long-term schizophrenia management. Internal FRs, such as supportive family relationships and coping capacities, and external resources, including social and professional support, may help caregivers reduce caregiving burden and facilitate effective caregiving. Previous studies have demonstrated that caregiving burden, social support, professional support, and family relationships are important factors associated with QoL among caregivers of individuals with schizophrenia (14, 41, 42). As a widely used measure of health-related QoL, the SF-36 captures caregivers’ physical and psychological well-being, which may be influenced by the availability of FRs. Therefore, we hypothesized that higher M-FRAT scores would be positively associated with SF-36 scores. Spearman correlation analysis were conducted to examine the association between M-FRAT and SF-36 scores. Linear regression analysis were additionally performed, with SF-36 scores specified as the outcome variable, to evaluate whether M-FRAT scores were associated with variations in caregivers’ QoL. Prior to all linear regression analysis, assumptions of linearity, homoscedasticity, normality of residuals, and independence of residuals were assessed using residual diagnostic plots and the Durbin–Watson statistic.

## 3 Results

### 3.1 Stage 1: content validity

#### 3.1.1 Characteristics of experts

The expert panel comprised eight members: two professors from a medical university, one psychiatrist, and five physicians from CHSCs, including two mental health doctors and three general practitioners (**Table 1**).

**Table 1:** Characteristics of the content validation experts (N = 8).

Variables	Category	N (%)
Gender		
	Male	2
	Female	6
Age (years)		
	<35	1
	35-45	5
	>45	2
Professional field		
	Professors in medical university	2
	Mental health doctors in hospital	1
	Mental health doctors in CHSCs	2
	General practitioners in CHSCs	3
Highest degree		
	Master’s degree	5
	Doctoral degree	3
Professional title		
	Mediate grade title	3
	Associate senior grade title	3
	Senior grade title	2

#### 3.1.2 Content validity

The I-CVI ranged from 0.88 to 1.00, and the S-CVI/Ave was 0.97, indicating excellent content validity of the M-FRAT.

### 3.2 Stage 2: item analysis, reliability, and construct validity

#### 3.2.1 Characteristics of caregivers

Of the 322 caregivers invited, 304 completed the survey (response rate: 94.4%). Non-participation was primarily due to stigma or reluctance to disclose information. Caregivers ranged in age from 19 to 94 years (mean ± SD = 59.30 ± 13.41); 44.7% were spouses and 71.0% had provided care for over 10 years (**Table 2**).

**Table 2:** Characteristics of caregivers of individuals with schizophrenia (N = 304).

Characteristics	N (%)	Characteristics	N (%)
Gender		Relationship with patient	
Male	148 (48.7)	Spouse	136 (44.7)
Female	156 (51.3)	Parent	96 (31.6)
Age (years)		Child	42 (13.8)
≤50	68 (23.0)	Brother or sister	20 (6.6)
51-60	86 (27.9)	Other Family relative	10 (3.3)
61-70	90 (29.8)	Caring years	
≥71	60 (19.3)	≤5 years	37 (12.2)
Education		6–10 years	51 (16.8)
Middle school and below	164 (53.9)	>10 years	216 (71.0)
High school degree	73 (24.0)	Insurance	
Bachelor degree (including associate degree) and above	67 (22.1)	Basic medical insurance	291 (95.7)
Marital status		Commercial insurance	1 (0.3)
Unmarried	14 (4.6)	Others	12 (4.0)
Married	257 (84.5)	Hours for caring patient per day	
Divorced	16 (5.3)	< 6 hours	173 (56.9)
Widowed	17 (5.6)	6–12 hours	83 (27.3)
Employment status		> 12 hours	48 (15.8)
Employed	74 (24.3)	Residence	
Retired	110 (36.2)	Rural	170 (55.9)
Unemployed	35 (11.5)	Urban	134 (44.1)
Others	85 (28.0)		

#### 3.2.2 Distribution of caregivers’ responses on the M-FRAT

Descriptive analysis showed that items related to economic and environmental resources had the highest proportions of “strongly disagree” responses. The highest proportions of “strongly agree” responses were observed for the items “2.5.8 Patients can receive free physical examination in the community once a year” (60.5%) and “1.5.1 Things that endanger the health of patients are placed in a safe location” (50.7%) (**Supplementary Material**).

#### 3.2.3 Distribution of family resources

**Table 3** presented the availability of FRs among families of individuals with schizophrenia. The total M-FRAT score averaged 163.01 ± 28.12 (range: 57-230). Domain-level scores and their corresponding ranges are also presented.

**Table 3:** Domain scores of the M-FRAT.

Dimensions	Mean ± SD	Range
1.1 Financial support	11.04 ± 2.74	3-15
1.2 Psychological and spiritual support	32.44 ± 6.33	8-40
1.3 Medical treatment support	12.35 ± 2.68	3-15
1.4 Information and education support	10.34 ± 2.93	3-15
1.5 Structural support	8.17 ± 1.59	2-10
2.1 Social resources	17.83 ± 4.71	5-25
2.2 Cultural resources	6.91 ± 2.27	2-10
2.3 Economic resources	18.55 ± 6.37	7-35
2.4 Environmental resources	9.87 ± 4.48	4-20
2.5 Medical resources	35.52 ± 7.70	9-45

#### 3.2.4 Item analysis

The high-score group (n = 84) had a minimum score of 178, and the low-score group (n = 84) had a maximum score of 147. The CR values of all item ranged from 4.420 to 17.372, all exceeding 3.000 (*p* < 0.001), indicating significant item discrimination.

Items 1.1.1 (*r* = 0.299) and 2.3.7 (*r* = 0.284) were slightly below 0.300, but deleting them did not affect Cronbach’s α, suggesting they could be retained. The correlation coefficients between the remaining item scores and the overall scale score ranged from 0.301 to 0.729 (*p* < 0.001).

#### 3.2.5 Reliability

The M-FRAT showed high internal consistency (Cronbach’s α = 0.938). As shown in **Table 4**, nine dimensions demonstrated satisfactory internal consistency, whereas the structural support showed inadequate internal consistency and was considered for removal.

**Table 4:** Internal consistency of the initial M-FRAT dimensions.

Dimensions	Cronbach’s α
1.1 Financial support	0.825
1.2 Psychological and spiritual support	0.913
1.3 Medical treatment support	0.848
1.4 Information and education support	0.729
1.5 Structural support	0.117
2.1 Social resources	0.777
2.2 Cultural resources	0.962
2.3 Economic resources	0.819
2.4 Environmental resources	0.815
2.5 Medical resources	0.904

#### 3.2.6 Construct validity

(1) Structural validity

The KMO index was 0.891, and Bartlett’s Test of Sphericity yielded a value of 10578.515 (*p* < 0.001), indicating sufficient sampling adequacy and confirming the suitability for factor analysis. Using PCA, nine factors with eigenvalues greater than 1 were extracted following orthogonal rotation using the varimax method, accounting for 70.312% of the total variance.

In the initial factor analysis, items 1.5.1, 2.1.4, and 2.1.5 demonstrated cross-loadings, indicating that they were not clearly associated with a single underlying construct. Item 1.5.2 exhibited a loading below 0.50, and item 1.4.3 showed a cross-loading pattern across higher-order domains, suggesting conceptual ambiguity. These items were therefore removed, and the model was re-run. In the second iteration, item 2.3.4 showed a loading below 0.50, and items 2.1.2 and 2.5.1 loaded onto factors different from their theoretical assignments; these items were subsequently deleted. In the third factor analysis, item 2.3.7 also exhibited a loading below 0.50 and was removed.

Ultimately, the final version of the M-FRAT for families affected by schizophrenia comprised 37 items loading onto seven factors, which collectively accounted for 70.245% of the total variance and demonstrated excellent internal consistency (Cronbach’s α = 0.927). Factor 1, *Economic and Environmental Resources* (merging dimensions 2.3 and 2.4), comprised nine items; Factor 2, *Psychological and Spiritual Support*, eight items; Factor 3, *Medical Resources*, eight items; Factor 4, *Medical Treatment-related Support* (merging dimensions 1.3 and 1.4), five items; Factor 5, *Financial Support*, three items; Factor 6, *Social Resources*, two items; and Factor 7, *Cultural Resources*, two items, as shown in **Table 5**.

**Table 5:** Rotated component loadings and internal consistency of the revised M-FRAT.

Dimensions	Items	Factors						
		1	2	3	4	5	6	7
Internal FR Financial support α = 0.825	1.1.1 The family can provide the basic living expenses for the patient					0.853		
	1.1.2 The family is able to pay the patient’s medical expenses					0.869		
	1.1.3 Family members can pay patient social expenses as needed					0.606		
Psychological and spiritual support α = 0.913	1.2.1 Family members can appease/tolerate the patient’s negative emotions		0.822					
	1.2.2 Family members can listen carefully to the patients’ complaints		0.869					
	1.2.3 Family members can give patients the confidence		0.882					
	1.2.4 Family members can support the patient’s interests		0.557					
	1.2.5 When the patient encounters difficulties, the family can show their love		0.824					
	1.2.6 The patient participates in the decision-making of major or important family events		0.817					
	1.2.7 At least two people in the family can share the care for the patient		0.541					
	1.2.8 Family members often pay attention to the patient’s disease state		0.745					
Medical treatment-related support α = 0.829	1.3.1 Family members can accompany the patient to seek medical treatment				0.747			
	1.3.2 Family members can urge patients to take medication				0.776			
	1.3.3 Family members can seek the most appropriate medical resources for patients				0.831			
	1.4.1 Family members can provide educational information on care				0.584			
	1.4.2 Family members are able to help patients learn new knowledge or skills				0.559			
External FR Social resources α = 0.764	2.1.1 There are publicity agencies or organizations in the society or community that provide mental illness-related policies for the patient’s family						0.719	
	2.1.3 There is a mental illness prevention information platform in the community or region where the patient lives						0.744	
Cultural resources α = 0.962	2.2.1 The community has publicity related to mental illness to reduce discrimination against patients and families							0.663
	2.2.2 There is publicity about mental illness in the community to reduce the stigma of families							0.661
Economic and environmental resources α = 0.908	2.3.1 Patients can obtain financial subsidies through organized rehabilitation labor	0.817						
	2.3.2 Patients enjoy employment economic support	0.854						
	2.3.3 Patients with mental illness and other major physical diseases can enjoy special financial support	0.838						
	2.3.5 Children of patients receive corresponding subsidies in the process of education	0.770						
	2.3.6 The patient receives financial support from employers	0.751						
	2.4.1 Welfare guarantee for the living conditions of the patient’s family	0.660						
	2.4.2 There is an “elderly canteen” in the community where the patient lives	0.604						
	2.4.3 Patients can be admitted to nursing institutions when needed	0.635						
	2.4.4 Patients can obtain services from rehabilitation medical institutions when needed	0.649						
Medical resources α = 0.909	2.5.2 There are mental health doctors in community health service institutions			0.679				
	2.5.3 In the community, patients and their families have received family rehabilitation guidance in the past half year			0.820				
	2.5.4 Family-based behavioral therapy provided by community-based mental health doctors			0.834				
	2.5.5 Patients can receive free medicines with less side effects and targeted medication guidance			0.647				
	2.5.6 Community health service centers often organize health education and other activities			0.668				
	2.5.7 Technical guidance from mental health doctors in Community health service centers			0.829				
	2.5.8 Patients can receive free physical examination in the community once a year			0.690				
	2.5.9 Establish a community prevention and treatment network in the local community to reduce the family’s monitoring pressure on patients			0.755				

The PAF solution yielded a seven-factor structure consistent with the original PCA solution. FA-based parallel analysis also supported the retention of seven factors, with the observed eigenvalues of the first seven factors exceeding those derived from randomly generated datasets. The factor loading pattern was generally comparable with the PCA solution, with all items showing factor loadings above 0.40 (**Supplementary Material**).

(2) Hypothesis testing

(i) Known-groups validity

Known-groups validity was examined by comparing M-FRAT scores between rural and urban families. Although no statistically significant difference was observed in total M-FRAT scores between rural and urban families (p = 0.089), significant differences were identified at the domain level. Rural families reporting higher scores in the cultural resources domain than urban families (7.16 ± 2.16 vs. 6.59 ± 2.38, p = 0.030). No significant differences were found in the remaining domains as shown in **Table 6**. These findings provided partial support for the predefined hypothesis that FR profiles may vary across residential contexts.

**Table 6:** Comparison of M-FRAT total and domain scores between rural and urban families.

Domains	Mean ± SD		t	p
	Rural (n=170)	Urban (n=134)		
Total M-FRAT score	131.39 ± 24.41	127.01 ± 20.26	1.709	0.089
Financial support	11.28 ± 2.85	10.74 ± 2.58	1.720	0.086
Psychological and spiritual support	33.05 ± 6.66	31.66 ± 5.83	1.948	0.052
Medical treatment-related support	19.46 ± 4.33	19.42 ± 4.04	0.084	0.933
Social resources	6.82 ± 2.50	7.16 ± 2.06	-1.294	0.197
Cultural resources	7.16 ± 2.16	6.59 ± 2.38	2.184	0.030^*^
Economic and environmental resources	21.45 ± 9.76	20.56 ± 8.93	0.822	0.412
Medical resources	32.17 ± 7.53	30.90 ± 6.65	1.543	0.124

^*^ P<0.05.

(ii) Association between M-FRAT and SF-36 scores

**Figure 1:**
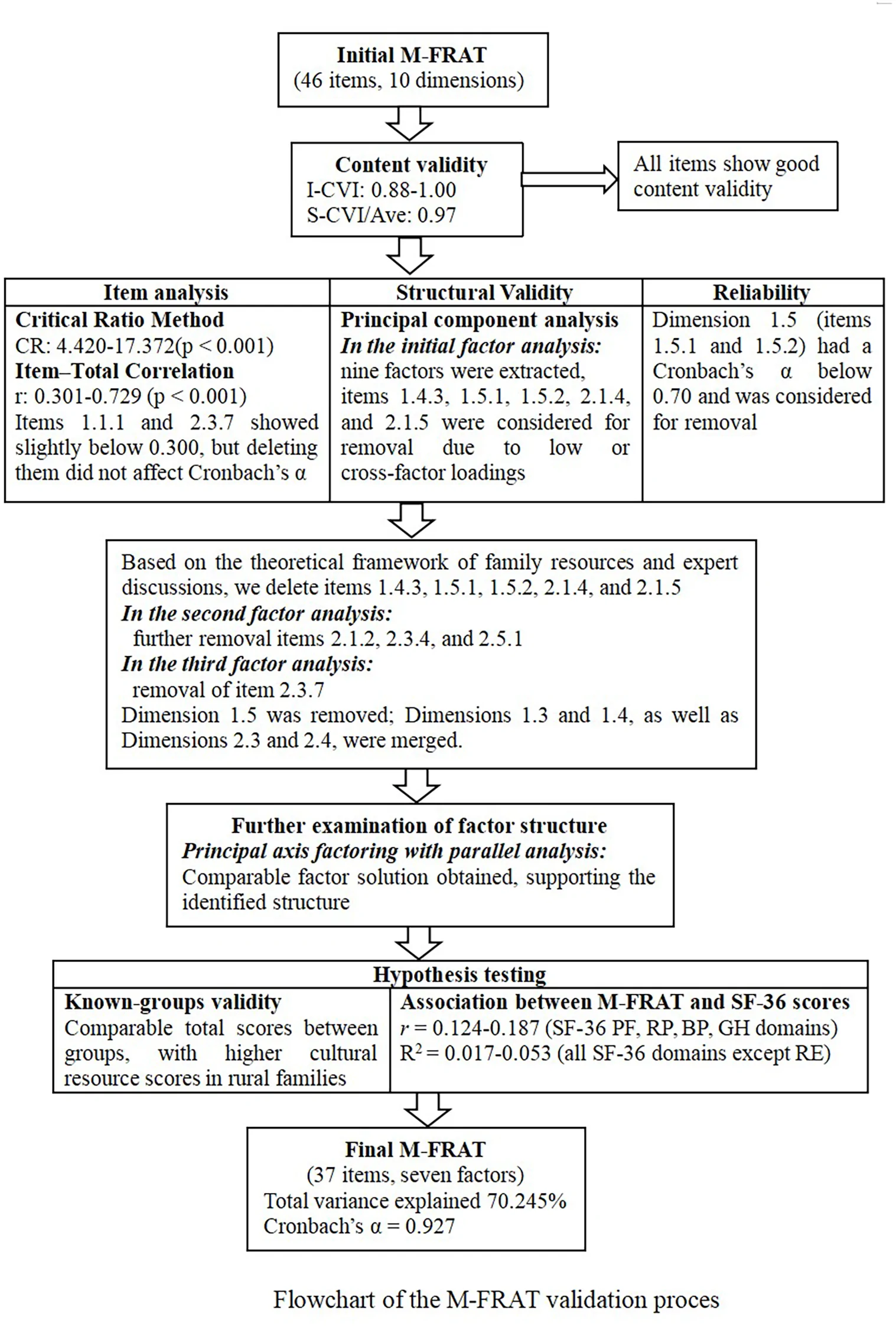
Flowchart of the M-FRAT validation process.

Among the eight dimensions of the SF-36, family caregivers demonstrated relatively low scores in GH, VT, and MH (**Table 7**). The final M-FRAT was positively correlated with the PF, RP, BP, and GH domains of the SF-36 (r = 0.124-0.187). Following confirmation that the assumptions of linear regression were adequately met, linear regression analysis indicated that higher M-FRAT scores were significantly associated with better outcomes across most SF-36 domains (R^2^ = 0.017-0.053), as shown in **Table 8**. An overview of the M-FRAT validation process was presented in **Figure 1**.

**Table 7:** SF-36 domain scores of caregivers.

Dimensions	Mean ± SD	Median
PF	78.16 ± 21.33	85.0
RP	58.14 ± 43.25	75.0
BP	72.44 ± 22.96	74.0
GH	57.83 ± 22.44	55.0
VT	57.63 ± 18.13	50.0
SF	71.46 ± 20.15	87.5
RE	62.17 ± 42.71	83.3
MH	58.62 ± 15.81	56.0

PF, Physical Functioning; RP, Role-Physical; BP, Bodily Pain; GH, General Health; VT, Vitality; SF, Social Functioning; RE, Role-Emotional; MH, Mental Health.

**Table 8:** Correlations and linear regression analysis between M-FRAT and SF-36 domain scores.

Independent variable	Dependent variable	Correlations analysis	Linear regression analysis				
		r	B	Std. error	Beta	t	P
M-FRAT	PF	0.154**	0.156	0.053	0.166	2.930	0.004^**^
	RP	0.175**	0.383	0.107	0.202	3.577	<0.001^**^
	BP	0.187**	0.232	0.057	0.23	4.103	<0.001^**^
	GH	0.124*	0.129	0.056	0.131	12.291	0.023^*^
	VT	0.071	0.126	0.045	0.157	2.771	0.006^**^
	SF	0.094	0.164	0.058	0.161	2.835	0.005^**^
	RE	0.032	0.169	0.108	0.090	1.571	0.117
	MH	0.081	0.123	0.039	0.177	3.122	0.002^**^

^**^ P<0.01, ^*^P<0.0.5.

## 4 Discussion

In this study, the M-FRAT was evaluated among family caregivers of individuals with schizophrenia in community settings in Beijing, providing preliminary evidence supporting its content validity, construct validity, and reliability. Higher M-FRAT scores were positively associated with better outcomes across multiple SF-36 domains. The tool contained 37 items encompassing seven domains: financial, psychological-spiritual, and medical treatment-related support within the family, and social, cultural, economic-environmental, and medical resources external to the family.

The M-FRAT demonstrated satisfactory psychometric properties for assessing FRs among families affected by schizophrenia in primary health care. Compared with previous scales (22–25, 43), the M-FRAT provides a more detailed and comprehensive assessment of FRs, thereby aligning more closely with the specific needs of families affected by schizophrenia. Based on the EFA results, a seven-factor structure demonstrated a stable and interpretable pattern, which was further supported by the comparable factor solution obtained from the PAF analysis. The structural support dimension was not retained in the final factor structure after psychometric evaluation. In terms of internal FRs, family caregivers gave priority to financial resources, medical information, and support from other family members, whereas structural support was less salient in their caregiving experience (44–46). This may be related to the fact that the participating family caregivers were primarily caring for individuals with schizophrenia in a stable phase, during which immediate environmental modifications or structural adjustments were less frequently required. The absence of a distinct structural support domain should therefore be interpreted within this clinical context. These findings suggest that structural support may be more salient at different stages of illness or in different caregiving contexts than as a stable, distinct dimension of FRs among families affected by schizophrenia. The merging of the economic and environmental resource domains into a single factor may reflect their close conceptual relationship as formal external resources. Economic resources mainly represent policy-based financial assistance, including rehabilitation subsidies, employment-related benefits, and educational support, whereas environmental resources reflect welfare provisions and access to community- and institution-based services. From the caregivers’ perspective, these resources may function as complementary forms of external support that jointly address the needs of both individuals with schizophrenia and their families. Consequently, caregivers may perceive them as part of an integrated support system rather than as distinct categories of support. In this scale, religious resources were not included, as most experts involved in the development process agreed that religious activities may not be appropriate for individuals with schizophrenia. This view is consistent with previous studies suggesting that religious activities may blur the boundary between reality and psychotic experiences, potentially exacerbating symptoms (47). In some cases, religious beliefs may also interfere with medication adherence or delay timely treatment (48). Future research may consider examining religious resources to better understand their role across diverse cultural contexts (49).

We found that caregivers reported relatively low levels of external FRs, especially economic and environmental resources. Similar findings have also been reported among family caregivers of people with schizophrenia in Nanjing, China (24). For internal FRs, support related to medical treatment-related information and financial support from family members or relatives was also relatively insufficient. Several factors may help explain these findings. First, limited awareness and inadequate public promotion of available services leave many families unaware of existing support resources (50). In Beijing, only 15% of families were aware of rehabilitation employment subsidies, and fewer than 10% had actual used them. In addition, complex and cumbersome application procedures further hinder access to support. Second, existing support services are often insufficiently tailored to the needs of families affected by schizophrenia (39). For example, many employment-related economic support require recipients to participate in work or entrepreneurial activities, making them difficult to access for people with schizophrenia (51). Third, stigma and discrimination associated with schizophrenia may discourage families from seeking help and participating in community activities, thereby reducing opportunities to obtain rehabilitation-related information and support (39, 52). Finally, the substantial caregiving demands associated with schizophrenia often constrain family caregivers’ ability to maintain stable employment, leading to reduced household income (5). As a result, financial strain often compels families to prioritize essential living expenses over rehabilitation-related services, further restricting access to supportive resources because of cost barriers (6). The known-groups analysis further suggested that residential context may influence specific dimensions of FRs. Although overall M-FRAT scores did not differ significantly between rural and urban families, rural families reported higher scores in the cultural resources domain than urban families. Therefore, interventions should consider the diversity of family resource patterns and promote affordable, accessible, and context-sensitive support services for families affected by schizophrenia.

Family caregivers of patients with schizophrenia in Beijing experience substantially reduced QoL, markedly lower than that of the general Chinese population (53). Preliminary evidence from hypothesis testing supported the expected association between FRs and caregivers’ health-related QoL. Higher M-FRAT scores were positively associated with SF-36 scores, although the strength of these associations was relatively moderate. This finding is theoretically plausible because FRs represent one of multiple factors influencing caregivers’ QoL, which is a multidimensional construct shaped by interacting physical, psychological, social, and environmental influences. Accordingly, the modest associations observed in this study do not diminish the relevance of the M-FRAT but rather reflect the multifactorial nature of caregivers’ QoL. From a clinical perspective, identifying and optimizing FRs may represent a valuable component of comprehensive strategies to support caregivers’ QoL. However, resource-oriented approaches should be integrated with broader interventions addressing caregivers’ complex needs. Caregivers of individuals with schizophrenia often experience substantial and persistent caregiving demands, which may lead to physical exhaustion, emotional distress, reduced opportunities for self-care, and the adverse effects of stigma and social isolation (54). In addition, limited awareness, availability, and utilization of healthcare and social resources may further undermine caregivers’ capacity to cope with these challenges. Therefore, FRs should be viewed as a modifiable component influencing caregivers’ health-related QoL within a broader ecological context. Routine assessment using the M-FRAT may facilitate the early identification of unmet resource needs and inform resource-oriented interventions as part of comprehensive caregiver support strategies.

Compared with existing instruments that primarily assess single dimensions, such as social support or family functioning, the M-FRAT provides a more comprehensive assessment of internal and external FRs tailored to families affected by schizophrenia. By identifying strengths and gaps in FRs, the tool may assist primary healthcare professionals in developing individualized family support plans, facilitating connections with appropriate resources, and promoting family-centered care, which may ultimately help reduce caregiver burden and support patient recovery. Future studies should further examine the applicability and utility of the M-FRAT in intervention studies and routine community mental health practice.

While this study provides encouraging preliminary evidence supporting the psychometric properties of the M-FRAT, several methodological considerations warrant attention when interpreting the findings. First, this study provided preliminary evidence supporting the structural validity of the M-FRAT. The seven-factor structure identified through EFA was further supported by the comparable factor solution obtained from PAF analysis, suggesting the robustness of the identified structure. However, further confirmation using confirmatory factor analysis (CFA) was not conducted because of sample size limitations. Future studies using larger independent samples should conduct CFA to evaluate the fit of the proposed factor model and further establish the structural validity of the M-FRAT. Second, because this was a cross-sectional study, test–retest reliability and responsiveness to change could not be evaluated and should be examined in future longitudinal studies. Third, although the M-FRAT demonstrated significant associations with the SF-36 in the hypothesized direction, these associations were modest, suggesting that FRs represent one of several factors contributing to caregivers’ health-related QoL. Fourth, participants were recruited exclusively from urban Beijing, and validation in more diverse geographical, cultural, and healthcare settings is warranted to enhance the generalizability of the findings (55). Finally, because both the M-FRAT and the SF-36 relied on caregiver self-report, the possibility of common-method bias cannot be entirely excluded.

## 5 Conclusions

The M-FRAT is a comprehensive caregiver-reported measure of FRs for families affected by schizophrenia and has demonstrated satisfactory validity, reliability and acceptability. The tool may enable primary healthcare professionals to systematically assess family needs in schizophrenia and inform targeted support to reduce caregiver burden and support patient recovery. Future research will validate the M-FRAT across a broader range of regions and cultural contexts in China to further assess its utility.

## Data Availability

The raw data supporting the conclusions of this article will be made available by the authors, without undue reservation.
