# Supplementary material for: Psychometric evaluation of a multidimensional family resource assessment tool for schizophrenia (M-FRAT) from caregivers’ perspectives in communities of Beijing, China
Source: Front Psychiatry. 2026 Sep 10;17:1818532. doi: 10.3389/fpsyt.2026.1818532 (PMC13601348; doi:10.3389/fpsyt.2026.1818532)
Supplement: Supplementary file 1 [file SupplementaryFile1.docx]

***Supplementary Material***

**Table 1**　The Initial Multidimensional Family Resource Assessment Tool for Schizophrenia (M-FRAT)

| The first-level indicators | The second-level indicators | The third-level indicators |
| --- | --- | --- |
| 1. Internal family resources | 1.1 Financial support | 1.1.1 The family can provide the basic living expenses for the patient |
|  |  | 1.1.2 The family is able to pay the patient's medical expenses |
|  |  | 1.1.3 Family members can pay patient social expenses as needed |
|  | 1.2 Psychological and spiritual support | 1.2.1 Family members can appease/tolerate the patient’s negative emotions |
|  |  | 1.2.2 Family members can listen carefully to the patients’ complaints |
|  |  | 1.2.3 Family members can give patients the confidence |
|  |  | 1.2.4 Family members can support the patient’s interests |
|  |  | 1.2.5 When the patient encounters difficulties, the family can show their love |
|  |  | 1.2.6 The patient participates in the decision-making of major or important family events |
|  |  | 1.2.7 At least two people in the family can share the care for the patient |
|  |  | 1.2.8 Family members often pay attention to the patient's disease state |
|  | 1.3 Medical treatment | 1.3.1 Family members can accompany the patient to seek medical treatment |
|  |  | 1.3.2 Family members can urge patients to take medication |
|  |  | 1.3.3 Family members can seek the most appropriate medical resources for patients |
|  | 1.4 Information and education | 1.4.1 Family members can provide educational information on care |
|  |  | 1.4.2 Family members are able to help patients learn new knowledge or skills |
|  |  | 1.4.3 Family members can provide information that help to rehabilitation |
|  | 1.5 Structural support | 1.5.1 Things that endanger the health of patients are placed in a safe location |
|  |  | 1.5.2 Convenient living conditions for patients |
| 2. External family resources | 2.1 Social resources | 2.1.1 There are publicity agencies or organizations in the society or community that provide mental illness-related policies for the patient's family |
|  |  | 2.1.2 There are specialized institutions or organizations to implement relevant policies for patients and families |
|  |  | 2.1.3 There is a mental illness prevention information platform in the community or region where the patient lives |
|  |  | 2.1.4There are caregivers mutual-help groups in the community/village where the patient lives |
|  |  | 2.1.5 When the patient's disease occurs, the neighborhood committee/community health service center/police can assist patient to ask for doctors |
|  | 2.2 Cultural resources | 2.2.1 The community has publicity related to mental illness to reduce discrimination against patients and families |
|  |  | 2.2.2 There is publicity about mental illness in the community to reduce the stigma of family caregivers |
|  | 2.3 Economic resources | 2.3.1 Patients can obtain financial subsidies through organized rehabilitation labor |
|  |  | 2.3.2 Patients enjoy employment economic support |
|  |  | 2.3.3 Patients with mental illness and other major physical diseases can enjoy special financial support |
|  |  | 2.3.4 Patients with severe mental illness enjoy free medical treatment in psychiatry |
|  |  | 2.3.5 Children of patients receive corresponding subsidies in the process of education |
|  |  | 2.3.6 The patient receives financial support from employers |
|  |  | 2.3.7 Caregivers are entitled to guardian allowance |
|  | 2.4 Environmental resources | 2.4.1 Welfare guarantee for the living conditions of the patient's family |
|  |  | 2.4.2 There is an "elderly canteen" in the community where the patient lives |
|  |  | 2.4.3 Patients can be admitted to nursing institutions when needed |
|  |  | 2.4.4 Patients can obtain services from rehabilitation medical institutions when needed |
|  | 2.5 Medical resources | 2.5.1 There are psychiatric hospitals around the patient's residence |
|  |  | 2.5.2 There are mental health doctors in community health service institutions |
|  |  | 2.5.3 In the community, patients and their families have received family rehabilitation guidance in the past half year |
|  |  | 2.5.4 Family-based behavioral therapy provided by community-based mental health doctors |
|  |  | 2.5.5 Patients can receive free medicines with less side effects and targeted medication guidance |
|  |  | 2.5.6 Community health service centers often organize health education and other activities |
|  |  | 2.5.7 Technical guidance from mental health doctors in Community health service centers |
|  |  | 2.5.8 Patients can receive free physical examination in the community once a year |
|  |  | 2.5.9 Establish a community prevention and treatment network in the local community to reduce the family's monitoring pressure on patients |

**Table 2**　Item-level distribution of caregivers’ responses on the M-FRAT

| **Items** | **Strongly disagree (%)** | **Disagree (%)** | **Neutral (%)** | **Agree (%)** | **Strongly agree (%)** |
| --- | --- | --- | --- | --- | --- |
| 1.1.1 | 3.3 | 6.3 | 34.5 | 33.2 | 22.7 |
| 1.1.2 | 4.9 | 7.9 | 31.9 | 27.0 | 28.3 |
| 1.1.3 | 3.9 | 6.9 | 29.6 | 31.6 | 28.0 |
| 1.2.1 | 2.6 | 2.0 | 19.1 | 30.9 | 45.4 |
| 1.2.2 | 3.0 | 3.3 | 19.1 | 32.2 | 42.4 |
| 1.2.3 | 3.0 | 2.0 | 18.8 | 32.9 | 43.4 |
| 1.2.4 | 3.0 | 1.6 | 26.0 | 34.9 | 34.5 |
| 1.2.5 | 1.6 | 2.0 | 17.8 | 33.6 | 45.1 |
| 1.2.6 | 3.0 | 2.3 | 18.4 | 37.2 | 39.1 |
| 1.2.7 | 10.5 | 9.5 | 18.8 | 29.3 | 31.9 |
| 1.2.8 | 1.6 | 1.0 | 15.5 | 34.5 | 47.4 |
| 1.3.1 | 1.3 | 4.6 | 15.1 | 28.6 | 50.3 |
| 1.3.2 | 4.3 | 3.3 | 13.2 | 28.9 | 50.3 |
| 1.3.3 | 1.6 | 9.2 | 19.4 | 32.2 | 37.5 |
| 1.4.1 | 3.3 | 9.2 | 25.0 | 31.3 | 31.3 |
| 1.4.2 | 12.5 | 11.5 | 28.6 | 26.6 | 20.7 |
| 1.4.3 | 12.8 | 14.1 | 27.6 | 26.3 | 19.1 |
| 1.5.1 | 10.2 | 2.3 | 14.5 | 22.4 | 50.7 |
| 1.5.2 | 0.7 | 2.3 | 18.4 | 37.8 | 40.8 |
| 2.1.1 | 13.5 | 3.9 | 24.0 | 31.9 | 26.6 |
| 2.1.2 | 4.3 | 0.7 | 14.5 | 31.3 | 49.3 |
| 2.1.3 | 13.2 | 8.6 | 23.4 | 32.6 | 22.4 |
| 2.1.4 | 22.0 | 8.6 | 23.7 | 23.7 | 22.0 |
| 2.1.5 | 17.8 | 4.3 | 17.4 | 30.9 | 29.6 |
| 2.2.1 | 9.5 | 5.3 | 33.9 | 32.6 | 18.8 |
| 2.2.2 | 10.2 | 5.3 | 33.9 | 30.6 | 20.1 |
| 2.3.1 | 40.8 | 15.1 | 22.4 | 11.8 | 9.9 |
| 2.3.2 | 42.8 | 13.8 | 23.0 | 10.9 | 9.5 |
| 2.3.3 | 45.1 | 10.5 | 22.4 | 13.5 | 8.6 |
| 2.3.4 | 22.0 | 6.9 | 16.4 | 18.8 | 35.9 |
| 2.3.5 | 48.4 | 16.1 | 18.1 | 8.6 | 8.9 |
| 2.3.6 | 50.3 | 15.8 | 16.4 | 8.2 | 9.2 |
| 2.3.7 | 6.6 | 4.6 | 20.4 | 23.0 | 45.4 |
| 2.4.1 | 50.3 | 16.4 | 17.1 | 10.9 | 5.3 |
| 2.4.2 | 42.8 | 9.2 | 18.8 | 18.4 | 10.9 |
| 2.4.3 | 33.9 | 7.2 | 24.7 | 21.4 | 12.8 |
| 2.4.4 | 33.9 | 9.5 | 26.6 | 17.4 | 12.5 |
| 2.5.1 | 4.3 | 5.6 | 19.4 | 35.9 | 34.9 |
| 2.5.2 | 3.9 | 1.0 | 13.5 | 35.2 | 46.4 |
| 2.5.3 | 7.9 | 2.6 | 14.8 | 38.5 | 36.2 |
| 2.5.4 | 7.9 | 0.7 | 18.1 | 34.9 | 38.5 |
| 2.5.5 | 7.6 | 0.7 | 17.1 | 32.9 | 41.8 |
| 2.5.6 | 12.8 | 4.6 | 28.3 | 29.6 | 24.7 |
| 2.5.7 | 11.5 | 1.0 | 20.4 | 33.2 | 33.9 |
| 2.5.8 | 4.3 | 1.0 | 11.8 | 22.4 | 60.5 |
| 2.5.9 | 9.2 | 0.3 | 16.8 | 34.9 | 38.8 |

**Table 3**　Factor loadings from principal axis factoring with promax rotation

| Items | Factor 1 | Factor 2 | Factor 3 | Factor 4 | Factor 5 | Factor 6 | Factor 7 | Uniqueness |
| --- | --- | --- | --- | --- | --- | --- | --- | --- |
| 1.1.1 |  |  |  |  | 0.912 |  |  | 0.216 |
| 1.1.2 |  |  |  |  | 0.973 |  |  | 0.151 |
| 1.1.3 |  |  |  |  | 0.508 |  |  | 0.543 |
| 1.2.1 |  | 0.858 |  |  |  |  |  | 0.256 |
| 1.2.2 |  | 0.976 |  |  |  |  |  | 0.197 |
| 1.2.3 |  | 1.022 |  |  |  |  |  | 0.190 |
| 1.2.4 |  | 0.486 |  |  |  |  |  | 0.490 |
| 1.2.5 |  | 0.827 |  |  |  |  |  | 0.239 |
| 1.2.6 |  | 0.840 |  |  |  |  |  | 0.331 |
| 1.2.7 |  | 0.454 |  |  |  |  |  | 0.583 |
| 1.2.8 |  | 0.702 |  |  |  |  |  | 0.348 |
| 1.3.1 |  |  |  | 0.801 |  |  |  | 0.273 |
| 1.3.2 |  |  |  | 0.786 |  |  |  | 0.377 |
| 1.3.3 |  |  |  | 0.955 |  |  |  | 0.192 |
| 1.4.1 |  |  |  | 0.495 |  |  |  | 0.546 |
| 1.4.2 |  |  |  | 0.536 |  |  |  | 0.418 |
| 2.1.1 |  |  |  |  |  | 0.681 |  | 0.382 |
| 2.1.3 |  |  |  |  |  | 0.697 |  | 0.417 |
| 2.2.1 |  |  |  |  |  |  | 0.768 | 0.152 |
| 2.2.2 |  |  |  |  |  |  | 0.791 | 0.113 |
| 2.3.1 | 0.827 |  |  |  |  |  |  | 0.326 |
| 2.3.2 | 0.890 |  |  |  |  |  |  | 0.258 |
| 2.3.3 | 0.862 |  |  |  |  |  |  | 0.262 |
| 2.3.5 | 0.777 |  |  |  |  |  |  | 0.451 |
| 2.3.6 | 0.727 |  |  |  |  |  |  | 0.481 |
| 2.4.1 | 0.612 |  |  |  |  |  |  | 0.606 |
| 2.4.2 | 0.509 |  |  |  |  |  |  | 0.513 |
| 2.4.3 | 0.647 |  |  |  |  |  |  | 0.390 |
| 2.4.4 | 0.596 |  |  |  |  |  |  | 0.491 |
| 2.5.2 |  |  | 0.658 |  |  |  |  | 0.477 |
| 2.5.3 |  |  | 0.883 |  |  |  |  | 0.275 |
| 2.5.4 |  |  | 0.914 |  |  |  |  | 0.248 |
| 2.5.5 |  |  | 0.598 |  |  |  |  | 0.553 |
| 2.5.6 |  |  | 0.646 |  |  |  |  | 0.352 |
| 2.5.7 |  |  | 0.864 |  |  |  |  | 0.218 |
| 2.5.8 |  |  | 0.708 |  |  |  |  | 0.555 |
| 2.5.9 |  |  | 0.760 |  |  |  |  | 0.427 |

Note: Applied rotation method is promax.

**Table 4**　Results of FA-based parallel analysis for factor retention

| Factor | Observed eigenvalue | Mean random eigenvalue Retained |
| --- | --- | --- |
| Factor 1* | 9.889 | 0.781 Yes |
| Factor 2* | 5.482 | 0.653 Yes |
| Factor 3* | 2.227 | 0.591 Yes |
| Factor 4* | 1.353 | 0.531 Yes |
| Factor 5* | 0.693 | 0.488 Yes |
| Factor 6* | 0.664 | 0.436 Yes |
| Factor 7* | 0.555 | 0.397 Yes |
| Factor 8 | 0.229 | 0.345 No |

Note: Factors were retained when observed eigenvalues exceeded the corresponding mean eigenvalues generated from random datasets.
